# COVID-19 and Vaccination Campaigns as “Western Plots” in Pakistan: Government Policies, (Geo-)politics, Local Perceptions, and Beliefs

**DOI:** 10.3389/fsoc.2021.608979

**Published:** 2021-04-23

**Authors:** Inayat Ali, Salma Sadique, Shahbaz Ali

**Affiliations:** ^1^Department of Social and Cultural Anthropology, University of Vienna, Vienna, Austria; ^2^Community Health Sciences, Peoples University of Medical and Health Science Women, Nawabshah, Pakistan; ^3^Independent Researcher, Islamabad, Pakistan

**Keywords:** COVID-19, polio, vaccination, local perceptions, (geo-)politics, rumors, (mis-)trust, mistrust vaccine preventable diseases VPDs

## Abstract

Vaccination encounters multiple context-specific challenges—socio-cultural, economic, and political—that substantially affect its uptake. Likewise, natural disasters and health emergencies considerably impact immunization endeavors, such as the coronaviurs 2019 (COVID-19) pandemic that has overwhelmed the entire world. It was already anticipated that the pandemic would severely affect Pakistan's vaccination programs due to interruptions in routine vaccination and the overstretching of healthcare systems. Consequently, there are anticipations of outbreaks of other vaccine-preventable diseases (VPDs). Yet empirical evidence is missing. Drawing on qualitative research, this article focuses on the impact of COVID-19 on routine vaccination programs in Pakistan. Our data come from a small village located in Pakistan's Sindh province where local people refused the routine polio vaccine that was stopped for a while, then resumed in July 2020. They suspected both the vaccine and COVID-19 to be a “Western plot.” We argue that these perceptions and practices can be seen against the backdrop of economic, socio-cultural, and (geo)political forces, which are encoded in “societal memory.” Not only is there a need to reverse the significant impacts of COVID-19 on routine vaccination by arranging supplementary immunization activities (SIAs), but also the government must deal with other pressing issues that affect the vaccination programs in the country.

## Introduction

Vaccination faces several challenges across the world that are context-specific (Nichter, 1995; Feldman-Savelsberg et al., 2000; Ali, 2020a,c). Among socio-cultural, economic, and political factors, natural disasters and health emergencies such as the coronavirus (COVID-19) pandemic considerably affect immunization endeavors (Ali, 2020c; World Health Organization, 2020). This pandemic has posed serious challenges to and interruptions in routine immunization activities around the globe, such as the need for physical distancing and the fact that COVID-19 has overwhelmed healthcare systems in many countries to the point where many vaccination programs have been temporarily halted (Ali, 2020d).

There is evidence that when vaccination is halted as resources are shifted elsewhere, vaccine-preventable diseases (VPDs) cause severe outbreaks, as was observed during the Ebola outbreak in West Africa (Masresha et al., 2020). Drawing on their risk-benefit analysis of routine childhood immunization in 54 African countries, Abbas et al. (2020) argue that the benefits to continuing routine childhood immunization programs outweigh the risk of COVID-19 contagion related to vaccination clinic visits. To continue vaccination, they suggest the use of necessary protective measures—physical distancing, personal protective equipment (PPE), and effective hygiene practices—to prevent COVID-19 transmission during vaccination administration encounters.

In this article, we argue that, both during the pandemic and in normal times, vaccination campaigns in low-resource settings face multiple challenges, especially when factors like (geo)politics are in play. For example, in Pakistan, socio-cultural, economic, and (geo)political factors significantly shape local perceptions and practices around both COVID-19 and vaccine administration (Abimbola et al., 2013; Ali, 2020b). Rumors and conspiracy theories have long been affecting vaccination programs in Pakistan (Abimbola et al., 2013; Khan and Chiau, 2015; Ali, 2020a,b).

During the pandemic's early days, it was rightly anticipated that COVID-19 containment measures would substantially affect routine vaccination programs. There were no empirical evidence for this, simply plausible assumptions. Yet by now, these assumptions have been verified (Ali, 2020b). It was also predicted that due to distinct socio-cultural, economic, and political factors, the impact of the pandemic on vaccination programs would be distinct in low-income countries like Pakistan (Ali, 2020b). There is also convincing evidence that one out of every two children has missed their routine vaccinations in Pakistan's Sindh province during COVID-19 (Chandir et al., 2020).

Chandir et al. (2020) have explored a 51% decline in overall immunization visits in the province that they claim are due to a reduction in the provision of immunization services, due to strict restrictions on the movements of vaccinators to do outreach, and to stock-outs resulting from disruptions in global manufacturing and supply chains, border closures, and general restrictions on local mobility (ibid.). On the demand side are the fear and hesitancy of parents to get their children vaccinated due to possible infection exposure, and to “myths” and misinformation about vaccination and rumors surrounding COVID-19, lockdown restrictions, unavailability of public transportation, and an increase in commuting costs (ibid.). In Pakistan, immunization activities have been significantly more affected in rural areas than in the Sindh province's urban areas (ibid.), where we conducted our research. In this article, we focus on government responses to COVID-19 and their effects on vaccination programs, and on myths and rumors that underlie local people's resistance to vaccination, and to government measures to control viral spread.

From the firsthand data we have obtained from a small village situated in rural Sindh, we learned that local people sent the “polio” vaccination teams back because they believed that both vaccines and the coronavirus are “Western plots.” What they refused were actually routine vaccinations for measles and Bacillus Calmette–Guérin (BCG), thinking that the vaccines offered were for polio. With the help of our past ethnographic data on vaccination (mainly collected in 2014) and our current research on COVID-19 in Pakistan, we present what factors affect people's choices to refuse and show resentment against vaccination and shape their anti-vaccination sentiments and explain why they consider these to be “Western plots.”

## Materials and Methods

This qualitative study obtained rapid information on the impact of COVID-19 on vaccination during August–September 2020. We divide this section into two parts for clarity: data collection (methodology) and the locale.

### Data Collection: Methodology

Since this is a rapid response study, Shahbaz Ali conducted 2 weeks of fieldwork during August–September 2020 in the locale where he had previously conducted his M.Phil. research on asthma. Due to this previous research in the same area and being part of Sindh province, he neither needed to take time to build the required rapport nor to learn the language, as Sindhi is his mother tongue.

Building on these advantages, Shahbaz conducted several group discussions using a method that lead author Inayat Ali has called *Kachahār*&#*x00131;̄* (Ali, 2020a). This method begs a brief explication. *Kachahār*&#*x00131;̄* is a local word that means discussions; ethnographically the *Kachahār*&#*x00131;̄* method as Ali has developed it is an adaptation of the “focus group” discussion, with the difference that the discussion is driven not by a researcher but by participants; it builds on an existing culturally recognized local social process for discussing and solving a particular problem.

Using purposive or convenience sampling and an interview guide, Shahbaz organized 10 *Kachahār*&#*x00131;̄on* discussions of 2–3 h each with almost 50 adult males at their *Otāq* (guest house for male members). The interview guide had four main areas: (1) local perceptions and practices around COVID-19; (2) local perceptions of vaccination; and (3) the underlying reasons for vaccine refusal.

The data gathered during this fieldwork included no personal health information but did include an overall description of experiences and perceptions of COVID-19, in which we found that these people refused routine vaccines such as BCG, thinking that, as previously noted, this was only the polio vaccine, and relating both COVID-19 and vaccination to the “Western” world. Thereafter, our focus was given to both phenomena—perceptions, and refusals. The *Kachahār*&#*x00131;̄* discussions took place in the Sindhi language. Although these village inhabitants speak Balochi as their mother tongue, they are also proficient in Sindhi, which is the lingua franca of Sindh province.

With the permission of interlocutors, some discussions were audio-recorded. After transcribing the data into English and examining them using thematic analysis, we analyzed this data by reading the entire dataset several times to identify relevant themes, followed by listing, summarizing, reviewing, and refining these various themes. Our analysis addressed the following questions: (1) How do local people perceive COVID-19, and what practices do they perform to deal with it? (2) What are local perceptions of vaccination and why do people refuse it? (3) Why do people see COVID-19 and vaccination as “Western plots?”

Shahbaz Ali wore a mask during the *Kahchāri* sessions, whereas the village participants did not wear masks though they did maintain physical distance. To obtain the interlocutors' consent, Shahbaz informed them about the aims of the study.

### The Locale

The setting of this study is thoroughly described by second author Shahbaz Ali in his previous research (Ali, 2018). Briefly, the village is located in a desert area of Sindh province. With a population total of 150 households, it lies at a distance of around 70 km from the third-largest city of the province, called *Sakhar*. Most of the inhabitants are engaged in animal husbandry, agriculture, and daily wage labor. Some are government employees, such as primary school teachers. Most people are not formally educated, and the highest formal education degree is “master” (16 years of education). The education rate differs significantly gender-wise, as more men have formal education than women.

Many inhabitants do have religious education, as they can read the Holy Quran and perform *Namaz* (prayers). The village has neither health facility nor proper sanitation. Almost every inhabitant of this village perceives health and illness as predetermined and an act of God or of fate (*Qismat*). They practice medical pluralism: (a) home remedies that have roots in Unani medicine and Ayurveda; (b) verbal healing that includes prayers, offerings, and chants of specific verses of the Holy Quran; (c) visiting sacred places such as shrines of saints; (d) consulting a *Hakeem* (an herbalist who practices Unani or Ayurveda systems); (e) visiting a biomedical facility. This health-seeking behavior further differs in terms of the disease's etiology, a person's gender, and the family's economic situation, and access to healthcare facilities all of which affect people's choice to ignore a disease or to utilize a specific healthcare system. In the following sections, we present overviews of COVID-19 and vaccination programs in Pakistan as background and context for our study results.

## COVID-19 in Pakistan: An Overview

Pakistan reported the first infection of COVID-19 in two men who returned from Iran on February 26. Over time, infections increased; at the end of March 2020, the country reported around 1,400 people infected by the virus and 11 deaths. By 11 June 2020, the virus had infected around 120,000 people and caused over 2,000 deaths. By 10 December 2020, the virus had infected approximately 430,000 people, out of which over 8,600 have “officially” died (Johns Hopkins University, 2020). This may seem like a very small number of deaths out of so many cases; in other work, first author Inayat Ali (in press) has shown how the Pakistani government may be fabricating these numbers in order to make it appear that they are doing an excellent job of coping with COVID-19.

To contain the virus, “flatten the curve,” and safeguard public health, the Pakistani government has implemented several measures. At the beginning of the pandemic, flights to and from China, then Iran, Qatar, and Italy were suspended (Ali et al., 2020). Due to the unavailability of test kits in the country, the country sent specimens to China and the USA, and later on, imported 1,000 kits from China (Ali et al., 2020). On 13 March, when only around 30 people were infected, the government closed educational institutions and the border with Afghanistan and Iran and opened a quarantine camp for COVID-infected people at the Pak-Iran border (Khan, 2020). Afterward, the country banned congregations, including conferences and gatherings. On 17 March, the country's Prime Minister ruled out the option of lockdown while considering that 97% of patients recover (Ali et al., 2020), but the government of Sindh province implemented a lockdown anyway. Later on, the federal government announced a countrywide lockdown; deployed security forces to enforce COVID-19-infected people's entry into quarantine centers, of which more had been established; invoked Section 188 of the Pakistan Penal Code for violations; shut the markets; monitored inter-provincial borders; created a Corona Relief Tiger Force to educate people about the critical consequences; distributed food items among daily wage laborers, and approved a PKR1.2 trillion economic relief package (Ali and Ali, 2020; Ali et al., 2020). On the 9th of May 2020, the government lifted the countrywide lockdown, despite the fact that the wealthy, “elite” population wanted to extend it; and introduced and implemented a “smart” lockdown to place only virus hotspots under lockdown (Ali and Ali, 2020; Ali et al., 2020). Under this smart lockdown, educational and training institutions, restaurants (except for take-away), marriage halls, cinemas, business centers would remain shut. Sporting, social, and religious events were also banned.

Currently (as of December 16, 2020), “smart” lockdown is still under operation, yet many people are organizing marriage ceremonies. Moreover, there are specific socio-cultural, economic, and political factors that create a conducive environment for the virus to exert severe consequences in the country (Ali and Ali, 2020); we detail some of these below.

## Vaccination in Pakistan: Backdrop

Vaccination began in Pakistan in the 1970s, after the country signed the Charter with the United Nations (UN) to contain and eliminate various communicable diseases. Commencing the Expanded Program on Immunization (EPI) in 1976 on a pilot scale, it was extended across the country in 1978 (Ali, 2020a). Although the EPI in the beginning intended to protect children aged 0–11 months against six contagious diseases—childhood poliomyelitis, tuberculosis, diphtheria, tetanus, pertussis, and measles—to reduce child mortality and morbidity, over time, new vaccines were introduced, such as hepatitis B in 2002, Hemophilus influenza type b (Hib) in 2009, pneumococcal vaccine (PCV10) in 2012, and inactivated polio vaccine in 2015 (Ali, 2020a). Moreover, vaccinations are also given to pregnant women.

The EPI follows a top-down approach: federal, provincial, district, sub-district, and Basic Health Unit (BHU) levels. There are facilities for maintaining the cold chain at all these levels. The Executive District Officer (EDO) works as the district head and is responsible for receiving the vaccines. Also, there is an EPI focal person who is responsible for all duties, from storage to distribution. To ensure the vaccinations, the monitoring teams visit the field and interview the target group—parents. The frequent questions include: (a) Does a vaccinator visit your village to vaccinate your children? (b) Could you please show us the vaccination card, if your child is immunized? Moreover, the card shows, the vaccination status of that child in the absence of the card, the BCG scar is checked and considered as proof the child was vaccinated.

Each union council (UC) contains a BHU functioning under a medical officer (MO). Moreover, as a month starts, vaccinators of every BHU visit the district level office to receive vaccines according to the target population. A vaccinator maintains a stock register, which encompasses all daily records of a vaccine—the amounts received, used, and remaining. The MO supervises the entire process. At a UC level, the vaccine is stored in the respective BHU at a controlled temperature and then distributed among vaccinators according to the due and defaulter list for the vaccination.

### Introducing “E-Vaccs”

For making the vaccination program more “efficient” and “effective,” primarily through overcoming the problems related to inadequate geographical coverage and insufficient performance of field vaccinators, the EPI of *Punjāb* has revisited its strategies via adding smartphone technology: a digital system called “E-Vaccs” (electronic-vaccination) for ensuring the vaccinators' attendance in the field (Ali, 2020a). Following the same methods of e-health for using information and communication technologies (ICT) via the internet, the system comprises a smartphone application to record real-time immunization coverage in a centralized database. The application was introduced in June 2014 in Punjab's four districts as a pilot project and was implemented across the Punjab's districts by October 2015. The introduction of smartphones with GPS trackers is an innovative idea purposing the improvement of vaccinators' attendance in the field for ensuring vaccination to every child.

After introducing the E-Vaccs, the vaccinators' attendance dropped significantly, from 97 to 54%, which appeared a critical development due to a few probable reasons. First, the technology was new and perhaps too advanced for many vaccinators, who were unable to understand and use it efficiently. Second, vaccinators considered that nothing significant would happen if they did not mark their attendance on it, as it has been in the past. Third, they showed resistance to this application because it would increase their accountability at the district level. They would no longer be able to mark themselves present by proxy; therefore, they wanted to make this move fail. Forth, some of them “lost” their smartphones and were using that as an excuse.

However, effective use of this app increased to 94% after fixing the problems cited above, because the vaccinators received training at the district level to impart the skills and knowledge to operate the phones appropriately; regular monitoring commenced; and the absentees received show-cause notices. Except Sindh province, this smartphone technology was launched in the Khyber Pakhtunkhwa (KPK) and *Baluchistān* provinces in 2016.

Moreover, another pilot program—*Har Zindagi*—was operational in the *Punjāb* province's two districts, *Sāhiwal* and *Sheikhupurā*, and will be implemented in the whole province and the country once it is shown to be successful (Sarwar, 2017). Working as a tool for maintaining the vaccination record at the household level by the parents, this new immunization card has the color and design akin to the country's passport (green) with the purpose of parents' intentional respect and vigilance. It has a Near-Field Communication tag inside, which enables real-time data sharing between the E-Vaccs application of the vaccinator and the card in the smartphone once both are tapped together. This application will work as an alarm through Robo-calling and a short message service (SMS) for vaccination dates for the parents.

One genuine criticism of this technology-driven initiative could be that it implies that everyone in Pakistan has a smartphone, but most of the villagers do not have one. Yet there can be multiple reasons behind difficulty of use, such as economic affordability, low or no electricity to charge phones, and low internet bandwidth speed. Thus, it is essential to ask, what provisions are made for those who do not have and cannot afford a smartphone nor a means to charge one? So far, there are no visible efforts of the government to address these issues.

### Failing to Meet Vaccination Benchmarks

Pakistan still needs to achieve the expected benchmarks. The World Health Organization (WHO) accepts that despite the government's efforts and the WHO's noteworthy partners, the country has yet to meet the immunization indicators (World Health Organization, 2019; Ali, 2020a). The primary goals of eradicating polio, measles, and neonatal tetanus remain unachieved thus far. We note that although polio has been eradicated in most of the world, there are still recurrent polio outbreaks in Pakistan due to lack of full vaccination coverage. Numerous outbreaks of measles, pertussis, and diphtheria in several parts of the country are further evidence of poor routine coverage.

Moreover, there are substantial issues in cold chain maintenance, lack of skilled vaccinators, and weaknesses in the catchment areas in terms of distance and geographical location (Khan and Chiau, 2015). These factors, which include people's perceptions as described below, significantly affect vaccine uptake. For instance, in September 2015, the *Dawn* newspaper published a report: “Over 75,000 children in Sindh never received polio vaccine” (Mansoor, 2015). According to this report, around 440,000 of the country's children never received the polio vaccine, and out of them, 56% are in Baluchistān, 17% in Sindh, 14% in the Federally Administered Tribal Areas (FATA), and 12% in KPK. These numbers accurately demonstrate the state of vaccination in the country, though the figures are about polio vaccination. If polio vaccination is in such a critical state despite securing enormous attention from the governmental and global stakeholders, then one can predict that there is something critically wrong with the country's entire immunization program.

According to Mushtaq and colleagues, from 2003 to 2006, polio was transmitted to approximately 24 otherwise polio-free countries, causing around 1,400 cases, most of which had originated in Pakistan (Mushtaq et al., 2015). In 2007, Australia reported a poliovirus infection in a man who had traveled there from Pakistan (Stewardson et al., 2009). Likewise, the strains of the poliovirus in cases identified in China, Egypt, and Palestine during January 2012, December 2012 and March 2013, respectively, were also traced back to Pakistan (Luo et al., 2013). This led, the Saudi government to make polio vaccination mandatory for Pakistani travelers to Saudi, especially for the Hajj and Umrah^1^. In 2011 and 2020, the WHO also made it compulsory for Pakistanis who would like to travel abroad to show a vaccine card at airports (Ali, 2020a; Chaudhry, 2020).

Pakistan is one of the two polio-endemic countries; the other is its neighboring Afghanistan. Both have collectively contributed 85% of recent polio cases globally (Ali and Ali, 2020). As noted above, in 2019 the country reported around 150 people infected with poliovirus, and by April 2020, there were around 30 newly infected people (Global Polio Eradication Initiative (GPEI)., 2020). The wild poliovirus is still prevalent in Pakistan and Afghanistan. The country also has been reporting new measles outbreaks (Ali, 2020a,b). Maternal and neonatal tetanus also prevails in the country (Iqbal et al., 2020).

All these VPDs reveal substantial issues in the Expanded Program of Immunization (EPI). As described above, low vaccination uptake results from the problems related to administering vaccines, and from people's distrust of vaccines, of the government, and of the global stakeholders. The phenomenon of vaccine refusals is complex and related to “the histories, politics, and social structures” (Closser et al., 2016). Also refusals are associated with the government's failure not to meet other responsibilities toward people (Closser et al., 2015). Parents' refusals and resentment chose not to vaccinate children are strong in those areas where the Pakhtun population lives due to a dearth of requiring knowledge about vaccination, low income, and formal education levels, as well as the number of children per household (Shah et al., 2019).

Consequently, a growing number of children remain unvaccinated. The recent *Pakistan Health and Demographic Survey* revealed that only 51% of children in Pakistan had all age-appropriate vaccinations (National Institute of Population Studies (NIPS) [Pakistan] ICF, 2019). That means almost half of the children receive no vaccine according to their age.

### Vaccination During COVID-19: Global Worries About Vaccination Programs

COVID-19 is an emergent and continually evolving phenomena. As its prevalence escalates in “developing countries,” it will significantly affect the weak healthcare systems of low-income settings. Various health initiatives, including ongoing vaccination programs, have been affected due to several interruptions in them resulting from the overstretching of healthcare systems (Ali, 2020b). The United Nations has expressed concerns about the millions of children who will not receive vaccinations against measles, diphtheria, and polio, and who will then be at critical risk of infection (United Nations News, 2020). During March 2020, the WHO's Strategic Advisory Group of Experts on Immunization (SAGE) recommended to all countries to suspend mass vaccination drives against all VPDs (Roberts, 2020). This suspension of vaccination will result in 78 million unvaccinated children for measles alone (ibid.). This aggregate would significantly increase if the remaining countries are counted or other VPDs are considered.

Besides measles, COVID-19 may substantially affect the longstanding Global Polio Eradication Initiative (GPEI). The GPEI had already directed countries to postpone their mass vaccination programs until the second half of 2020: these campaigns reach around 400–450 million annually (Roberts, 2020). WHO's Michel Zaffran—the head of GPEI—fears that the poliovirus will likely spread further to polio-free countries (Roberts, 2020).

## Study Results

In this section, we divide our results into two subsections: the local perceptions of COVID-19 and the local perceptions of routine vaccination.

### Local Perceptions of COVID-19

Local perceptions and practices play a pivotal role in whether or not the government measures can be effective. Here we describe these village people's knowledge, attitudes, and practices during the extraordinary event of COVID-19. Around the country, various, and distinct rumors have circulated regarding the existence of COVID-19 and its potential treatment, such as drinking green tea, shaving one's head, or performing certain rituals and prayers (Ali, 2020c). Based on their perceptions and practices, people in Pakistan can be divided into two groups: those who have ignored COVID-19 and those who become fearful and panicky after taking the virus seriously.

Similarly, conspiracy theories have revolved around the pandemic, considering it a “Western,” an “American,” or “Jews” pot (Ali, 2020c; Ali et al., 2020; Salma et al., under review)—a conspiracy of the Western world against the rest. For example, one interlocutor argued, “The West has created this virus as a conspiracy to affect us. The media also tell about it. Because of this creation, the entire world has been affected now.” These people think that the purpose of this “plot” is to sterilize Muslim women to control the population, as they believe that the “West” fears an increasing Muslim population (Ali, 2020a,b). They are not wrong in making that assumption—as evidenced by the protests in Europe against Brown and Muslim immigration, the tendency of white Americans to suspect that all Muslims may be “terrorists,” and of white people in general to consider themselves “superior” to all *Others* (Kaunert et al., 2015). This “superiority complex,” rooted in colonization and imperialism, both invokes white dominance and makes the “Others” to feel inferior and degraded, provoking (often invisible) resistance. Anthropology has long critically examined this *us* vs. *them* mentality as revealed in projects of “development,” “progress,” racism, colonization, and imperialism (Asad, 1973; Escobar, 1995; Acosta et al., 2020; Terror, 2020). The roots of this local resistance toward vaccination must be understood within this overall geopolitical context. From this perspective, we can understand how vaccination may be perceived at the local level not as a life-saving endeavor but as a “political project” (Ali, under review). In fact, in Pakistan the seeds of resistance against vaccination germinated as long ago as 1953, when many Pakistanis refused the government-initiated Family Planning Program, which they viewed within this context as “Western” effort to limit their reproduction. Thus, when the EPI was started across the country in 1978 after a pilot project in 1976, people suspected that the program was a tactic of the government and the Western world designed to ensure that fewer Muslims would be born (Ali, 2020a).

Returning to the village under study, we note that within this village, people have not followed the prescribed preventive measures, such as physical distancing and wearing masks. Yet, while going outside the village, especially to a nearby small town to buy common daily goods, they have been compelled to take these measures due to fear of the police. The following words of an interlocutor in his 50s describe this situation:

At our village, primarily at home, we have not followed measures, such as maintaining physical distancing and wearing masks strictly. However, when we went outside the village to buy food or other daily use items, we had no mask because it is expensive for us; we covered our nose and mouth with a handkerchief. This was especially due to a fear of police who were doing surveillance and putting a fine on the one without a mask. They were beating [people] as well as receiving money.

This man's words index the corruption that is syndemic in both the police and the Pakistani government. In its Corruption Perception Index for 2019, Transparency International reported that Pakistan stands at 120th out of 180 countries in terms of reducing corruption, revealing an increase in corruption compared with 2018 (Ali and Ali, 2020). This constant practice of corruption is one among the leading factors that inhibit the country's economic growth, making it vulnerable to foreign aid “dependency syndrome” (ibid.).

On the one hand, these local people considered the virus to be a “Western plot,” and on the other hand, as challenging and threatening. They were fearful of contracting it. During one group discussion, the participants unanimously opined:

We have been extremely anxious that the virus may affect us. Whoever coughed or sneezed in our village, we thought that the coronavirus had infected him/her. Thereafter, we tried to avoid that person. We have become suspicious. One person from our village started coughing and sneezing and asked us if this virus has infected him. At first, we ignored what he was saying, considering it as a joke. However, his cough and flu continued for a few days and then fever also occurred to him. After that, we become serious about him, since he was also asking us to bring him to a hospital. He was saying to us, “I am telling you that this virus has infected me. Please bring me to a hospital; otherwise, I may die.” His situation and his constant pleadings compelled us to make a distance from him as well as bring him to a doctor. After a few days of that severe onset, his family brought him to a doctor, who was also suspicious but gave him some medication. This person was not hospitalized because he had no severe issue with breathing. The doctor recommended him to maintain physical distancing and perform self-quarantine. Thus, when he returned to the village, this person was himself avoiding us to meet or talk while repetitively uttering to make us cautious, “Please go away from me, because I have coronavirus.”

### Local Perceptions of Routine Vaccination

In this section, we focus on how local people perceive routine vaccination during the ongoing pandemic and why they refused the polio vaccine and sent the mobile vaccination teams away (In Pakistan, there are two types of vaccination teams: mobile/outreach teams who visit each house to give the routine vaccines, and the fixed teams who sit at specific places to vaccinate children).

During COVID-19, on July 20, 2020, Pakistan resumed its vaccination campaign, which had been halted in March due to the pandemic, to reach around 800,000 children. With masks and gloves, vaccinators restarted vaccinating children (Reuters, 2020). Yet given the rumors and conspiracy theories revolving around COVID-19 in Pakistan (Ali, 2020c), inhabitants of this village also suspected this newly renewed routine vaccination program (Reuters, 2020). Their concerns are rooted in past vaccination drives as well as in the current rise of COVID-19.

First, these people refused the polio vaccine because they did not believe it would do their children any good. As one interlocutor stated, “As soon as the vaccination team arrived here, we promptly asked them not to vaccinate our children and return because they have been vaccinating our children for a long time. Yet, the health of our children does not improve. Many of them remain sick. Given that, what is the purpose of having our children vaccinated?”

Secondly, the local people linked the current vaccine drive to the ongoing pandemic. Their previously noted suspicions around COVID-19 also spilled over to vaccination. Considering the coronavirus's spread to be a “Western production,” they think that the vaccine is a product of the *Angraiz* (British)^2^. For example, one interlocutor stated, “It seems this virus is a product of the Angraiz. And if this assumption is true, then all medicines, including vaccines, are their production. Who knows what type are these vaccines, and what if we allow vaccinators to vaccinate our children and then our children die?”

Some interlocutors stated that vaccinators should give them a written document to mention that nothing bad will happen to their children after receiving the vaccine. Or, vaccinators should provide their Computerized National Identity Cards (CNICs) for the same purpose.

## Discussion: Vaccination Programs and COVID-19

Vaccination in many countries is a complex phenomenon. It has constantly remained under the spotlight due to its success stories as well to its failures (Feldman-Savelsberg et al., 2000; Blume, 2006; Closser, 2010; Fairhead and Leach, 2012; Greenough et al., 2017; Ali, 2020a). On the one hand, its advocates draw on the success story of the smallpox eradication and the prevention of thousands or millions of infections and deaths. This leads to worldwide continuous vaccine drives to vaccinate millions of children. On the other hand, as we have seen, its opponents suspect the ingredients and motives of its administrators. These suspensions and resentments result in a growing number of vaccine refusals and unvaccinated children. Although both perspectives can be found almost in every country, both are extremely prominent in low-income countries due to their lower educational levels and to the prevailing issues of mistrust in their governments and in global stakeholders, as we have described above.

Similarly, rumors and conspiracy theories have surrounded COVID-19 across the world due to uncertainty, anxiety and fear regarding a constantly evolving phenomenon of COVID-19 (Ali, 2020c; Ali et al., 2020; Jolley and Paterson, 2020; Romer and Jamieson, 2020; Uscinski et al., 2020). These narratives should be considered as “social phenomena” revealing interplay between various socio-cultural, economic, and (geo-)political factors (Ali, 2020c). These narratives would affect the upcoming vaccination against COVID-19, thus, it is necessary to understand the underlying factors (Ali, 2020a,b; Jolley and Paterson, 2020; Miller, 2020; Romer and Jamieson, 2020).

Likewise, the narrative of both COVID-19 and vaccination programs as “Western plots” has remained strong across the country, due in large part to the “fake” vaccination drive organized by the US in 2011 to locate Osama-bin-Ladin (Ali, 2020a,b) and to the American drones they see flying around, which they feel are “spying on them.” Owing to these various rumors and conspiracy theories linked to different past realities—such as British colonization or an almost decade-old “bogus” vaccination campaign—vaccination uptake in Pakistan has remained under par (Ali, 2020a). These strong perceptions and suspicions have had drastic consequences that include assaulting vaccination teams; over 100 vaccinators have been killed, despite the government provision of police escorts (Closser and Jooma, 2013; Ali, 2020a,b).

People also negotiate vaccines with the government. In return for having their children vaccinated, economically poor and marginalized people want to get something back from the government, especially economic support, which they rarely receive (Ali, 2020a,b). Both their perceptions and practices have their roots in the various socio-economic, economic, and political factors of the country, which play a pivotal role in the shaping of distinct imaginaries and actions (Ali and Ali, 2020). The views of local people of that selected village reflect such deeper and broader contexts: national and global. And their standpoints should be understood as the backdrop of this interplay between national and global contexts. These perceptions and practices of past abuses, injustice, and exploitation are preserved in “societal memory” (Ali, 2020a; Ali and Davis-Floyd, 2020).

The critical view to refuse the vaccine, to consider it dangerous to children's health, and to link it with the “West” also needs to be situated within the various forms of structured inequalities and inequities at play; as Famer argued, these various structures shape people's views and underpin the preparedness programs during any health emergency (Farmer, 1996). In other words, as a result of such disparities, the poor and rural receive less government attention and a lower quality of health care than the wealthy and the urban.

Studies show that people's perceptions of the COVID-19 pandemic as an “American” or “Western” plot impede polio eradication in Pakistan and create fertile ground for measles and other VPDs to flourish (Ali et al., 2020). Moreover, limited outdoor movement and lockdown policies might play pivotal roles in affecting the ongoing routine vaccination programs, as people fear that physical interaction may expose them to COVID-19 infection and so will not visit a healthcare facility to vaccinate their children. Likewise, many vaccinators also fear contagion and may refuse to perform routine vaccination (Ali, 2020b). Consequently, as we have shown, the COVID-19 pandemic is greatly affecting routine immunization coverage in Pakistan and augmenting vaccine hesitancy at local levels.

To control VPDs and to run effective routine vaccination programs thus require significant efforts. They necessitate a political, judicial, and economic infrastructure that provides a base to build a stable, secure, and proper society: a society that can thrive in ordinary times and effectively respond to extraordinary times. Not only vaccination programs, but the entire healthcare system is also intricately linked with corrupt governance, insufficient public works, injustice, ineffective social services, inadequate education, challenging subsistence patterns, and the natural environment. These factors affect the spread of infectious diseases and their courses in those affected (Farmer, 1996; Ali, 2020a; Ali and Ali, 2020). To improve this situation, effective, non-corrupt governance in the stated areas is essential to provide adequate healthcare. Political stability and governmental effectiveness provide immunization to “social and political bodies” (Scheper-Hughes and Lock, 1987) against corruption, social conflict, and instability, just as vaccination immunizes the physical body against numerous microorganisms. As infections weaken the physical body, so there are factors that weaken the entire socio-cultural and politico-economic systems of society, in which healthcare is also embedded.

## Limitations and Strengths of the Study

This study holds several limitations. Firstly, it involved only 50 male interlocutors who may not be speaking for the entire village, especially for its women. Secondly, it is a rapid response study based in part on only 2 weeks of fieldwork, though it draws on extensive previous fieldwork. Owing to these limitations, the results cannot be generalized. The great strength of this study is that it is likely the first to collect first-hand data from local people on how the pandemic has affected routine vaccination programs. From this perspective, the study has great scope, as we hope that it will trigger further research on this topic. An additional strength lies in its geographical focus, as vaccination in Pakistan remains under constant negotiation, and viruses such as polio and measles still cause significant outbreaks in that country.

## Conclusion and Recommendations

Despite the fact that one of the Sustainable Development Goals 2030 emphasizes the need to develop and distribute vaccines and medical research with easy access to cheap essential medicines and vaccines, especially for developing countries (World Health Organization, 2018), vaccination remains a pressing issue in low-income countries like Pakistan. This life-saving endeavor has become a politicized project, and due to that one of us calls for “an anthropology of vaccination” (Ali, under review). Moreover, because of these multiple factors working against effective routine vaccination programs, we can anticipate ongoing outbreaks of VPDs such as measles and polio, as well as refusals and resistance toward the COVID-19 vaccine in Pakistan and most likely in other countries as well.

Not only is there the need to reverse the significant impacts of COVID-19 on routine vaccination, but also it is crucial for the government to deal with the pressing issues that affect the vaccination program in the country. For this, effective, non-corrupt governance that equally attends to the rural poor's needs is essential to provide adequate healthcare. Again, *governmental stability, honesty, transparency, and effectiveness provide immunization to social and political bodies against corruption, social conflict, and political instability*. Just as infections weaken the physical body, so the infections of corruption and structural disparities weaken the governmental body and impede it from effective governance for all.

Undoubtedly, all efforts should be made to run the routine vaccination drives during these Covidian times to avert further challenges posed by other microorganisms. Yet, we argue that such programs will remain ineffective until the Pakistani people, especially the rural poor, are sufficiently educated in need for, the ingredients in, and the effectiveness of vaccines, as well as in the very real dangers of vaccination refusal. They say that their children get sick despite being vaccinated; they need to understand that lack of vaccination can lead their children to contract more VPDs. People need to feel that their government is on their side and is not perpetrating Western or American plots and conspiracies. Rather than what we would call “reactive” coping strategies, Pakistan and other countries like it need “proactive” programs to improve vaccine uptake and to effectively deal with future challenges. COVID-19 has proven that we all live in a global community; thus, adequate health care is both a national and an international obligation.

## Data Availability Statement

The datasets presented in this article are not readily available because, The article not only draws on the current qualitative and confidential data but it also draws on our long-term ethnographic data that cannot be shared. Requests to access the datasets should be directed to inayat_qau@yahoo.com.

## Ethics Statement

The studies involving human participants were reviewed and approved by Pakistan's National Bioethics Committee (reference No.4-87/NBC-471-COVID-19-09/20/). Written informed consent for participation was not required for this study in accordance with the national legislation and the institutional requirements.

## Author Contributions

IA contributed to the conceptualization, writing the first draft, analysis, revision, and validation. SaA and SS contributed to the revision, analysis, and validation. ShA contributed to the data collection, revision, analysis, and validation.

## Conflict of Interest

The authors declare that the research was conducted in the absence of any commercial or financial relationships that could be construed as a potential conflict of interest.

## Footnotes

^1^Hajj and Umrah are holy pilgrimages for Muslims, during which they travel to Makkah and Medina cities of Saudi Arabia to visit the holy *Qabā* and the shrine of the Prophet Muhammad.

^2^Due to its long tenure as a British colony, many people in Pakistan use the generic word *Angraiz* for every person with white skin from the West. For instance, in common discourse, they rarely distinguish between a person from Austria and one from the United Kingdom.
